# Psychological Variables Explaining the Students’ Self-Perceived Well-Being in University, During the Pandemic

**DOI:** 10.3389/fpsyg.2022.812539

**Published:** 2022-03-22

**Authors:** Laura Nicoleta Bochiş, Karla Melinda Barth, Maria Cristina Florescu

**Affiliations:** Department of Science Education, Faculty of Social Humanistic Science, University of Oradea, Oradea, Romania

**Keywords:** well-being, openness, personal autonomy, academic motivation, student

## Abstract

**Introduction:**

In the coronavirus disease 2019 (COVID-19) pandemic, Romanian universities switched to emergency relocation and online education, with students experiencing a sense of isolation, which affected their well-being, pace and normal learning style, relationships with other colleagues, and Professors. Beyond the technological obstacles that have arisen in learning, the aim of this study is to highlight the psychological variables that are associated and that explain the self-perceived well-being of students, in university, in the pandemic. The psychological variables studied were the following: the level of openness and personal autonomy, as personality traits of students but also the mechanisms for regulating their academic motivation.

**Method:**

We used a questionnaire-based survey, wherein all four research instruments had been validated and adapted to the investigated population. The subjects were BA and MA students at the University of Oradea, Romania (*N* = 150), the majority being females (95.5%) with the age range of 27 years old. Pearson Correlation and Multiple Linear Regression were used to test the two hypotheses.

**Results:**

Research data obtained in the correlation analysis, point out association relationships with moderate and high effects size, between positive attitude toward self, others and student life and: openness to learning, openness to aesthetics, behavioral autonomy, cognitive autonomy, intrinsic motivation, and identification motivation. Furthermore, in regression analysis, it was revealed that regarding the variance of results concerning students’ self-perceived well-being in university (positive attitude toward oneself, others, and student life), it contributes both of students’ personality traits (such as openness and personal autonomy) and their intrinsic motivation and identification motivation.

**Conclusion:**

The fundamental conclusion of the research is that, although the personality traits of students explain in a higher percentage the variability of results in students’ self-perceived well-being (in terms of positive attitude toward self, others, and student life), motivation regulation mechanisms play an important role, especially in the conditions of online activities. The results have direct implications for the work carried out in universities. The educational policies developed by specialists and government will have to emphasize the ways of forming resilient student communities in periods of sudden transition and adaptation to change which take place in education and society.

## Introduction

The key constructs addressed in this article, i.e., well-being, motivation, and personality, are at the heart of the concerns of researchers in various branches of psychology. In fact, due to the changes that took place in all sectors of activity, in the coronavirus disease 2019 (COVID-19) pandemic, the researchers emphasized the importance of identifying protective and risk factors, in relation to the balanced development of people and well-being (Robu and Tufeanu, 2020). Globally, the COVID-19 pandemic has represented not only a major medical and economic crisis but also a psychological one, being associated with declining levels of subjective well-being (SWB) (Akbas et al., 2021; Rodideal and Marinescu, 2021; Zacher and Rudolph, 2021).

The factors that influence the dynamics of well-being were studied by Lyubomirsky et al. (2005), who show that 50% of the variance of well-being is due to genetic factors, 10%, to circumstantial factors (micro and macro social), and the remaining 40%, to the variance of the well-being of the intentional activities. However, in the context of the pandemic, the percentages could be reconsidered and reanalyzed, either by certain categories of participants (for example, by groups of students) or in certain specific situations, such as the transition to online education. This is because the rapid and widespread transition to online education during the COVID-19 pandemic disrupted intercollegiate relationships and student learning routines, led to less familiar instructional methods, and created technological barriers to online student learning (Huang and Zhang, 2021). They do not have access to all tools and learning materials in the virtual environment or students simply did not participate in online courses (Yusuf and Jihan, 2020). Additionally, is the fact that in online learning, pupils and students face difficulties in memorizing and concentrating (Aftab et al., 2021). In addition to the difficulties of accessing and using technology to adapt students to online education, research has also shown that its sudden implementation has had serious consequences for students’ SWB (Hung et al., 2020; Lyons et al., 2020). It is known that social isolation, during the COVID-19 pandemic, has a negative effect on their well-being due to the increased emotional lability that people face in emergencies (Ivantchev and Stoyanova, 2021). Worryingly, pandemics have seen an increase in mental disorders (e.g., anxiety, depression, loneliness) among pandemics (e.g., Besser et al., 2020; Cao et al., 2020; Azzi et al., 2021; Islam et al., 2021; Liu et al., 2021).

Although researchers’ interest in people’s well-being increased significantly during the pandemic, less attention was paid to the study of internal psychological variables that explain the well-being of university students. Such studies are needed because researchers also point out that student well-being and learning may continue to be influenced by future uncertainties and modified pandemic and post-pandemic teaching or training programs (Aftab et al., 2021).

In the elaboration of this study, two of the research directions followed in the field were considered, directions related to the psychological variables that can contribute to the well-being of people, in general, and of students, in particular. The first of these considers the conclusions reached by researchers regarding the association between SWB and personality variables. For example, Muntele Hendreş (2021) highlighted that an important moderating factor in people’s happiness is their personality. The author points out that although people tend to react emotionally similar to identical events, the intensity and duration of their reactions are influenced by personality. Other research studies have highlighted the fact that individual personality differences play a key role in the relationship of association with the psychological well-being perceived by the population during the pandemic (Rossi et al., 2021; Simuţ et al., 2021). The second direction of our research was initiated by Deci and Ryan (1985, 2000). In the theory of self-determination, they say that the levels of motivation can be explained by a continuum of the levels of self-determination. On this continuum, three levels of motivation can be differentiated, namely: motivation, extrinsic motivation, and intrinsic motivation. Of these, intrinsic motivation is considered to contribute most to a person’s development and well-being. The explanation lies in the fact that people nurture, from an early age, the desire to be stimulated cognitively, socially, and emotionally, to learn and to use their potential (Deci and Ryan, 1985, 2000). However, recent studies have shown that maintaining intrinsic motivation in difficult times, including pandemics, can be difficult to achieve (Whitfield et al., 2021). Therefore, we consider it necessary to identify those mechanisms for self-regulation of students’ academic motivation, which correlate and explain the variance of their well-being in university, in a pandemic.

More detailed references on the key concepts studied, on the results of research related to the topic of our study, and the gap in the literature on internal psychological variables that influence the well-being of students in a pandemic will be highlighted in the next section.

## Literature Review

### The State of Well-Being and the Variables Related to Personality

#### Well-Being – In and Beyond the Conceptual Framework

The delimitation of the conceptual framework for well-being was presented by Ryan and Deci (2001) through the prism of two perspectives: hedonic and eudaimonic. The hedonic perspective on well-being pursues the level of happiness of a person, and the eudaimonic one pursues the optimal functioning level of the individual (Ryan and Deci, 2001). The most well-known approach to the study of well-being, from a hedonic perspective, is that of SWB. Several authors (Diener et al., 1985, 2009; Lyubomirsky et al., 2005) believe that people experience a high level of SWB when they feel more pleasant emotions and less unpleasant, and when they are satisfied with their own life. Thus, SWB requires both, an affective and a cognitive component, one of evaluating one’s own life.

From a eudaimonic perspective, well-being is understood as *psychological well-being* (PWB). Ryff (1989) shows a multidimensional approach to it, an approach that includes: autonomy, personal growth, self-acceptance, purpose in life, mastery, and positive relationships. In just a few words, in the eudaimonic sense, PWB can be characterized by the effort to achieve intrinsic goals and values, autonomous behavior, and the state of being and acting consciously in accordance with what is happening at a certain moment.

Understood as a multidimensional construct, Robinson et al. (1991) and later Adams et al. (1997) studied well-being starting from the evaluation of six specific areas of human functioning: (1) physics, (2) spiritual, (3) psychological, (4) social, (5) emotional, and (6) intellectual. Following the line of these works of research, in Romania, G. Roşeanu, and R. Răşcanu adapted and validated the instrument that measures the well-being of students in 2008. They ask that the answers to the items related to the six specific areas should be taken into consideration when evaluating well-being, and the presentation of the results be highlighted on three subscales: (1) positive attitude toward self, world, and life; (2) negative attitude toward self, world, and life; (3) state of physical health. Thus, in our research, the concept of the well-being of students refers rather to positive emotions and attitudes versus negative ones, to physical health, i.e., to SWB, and not to clinically diagnosed mental health disorders.

But in recent decades, the interest in studying the subjective and psychological state of well-being has extended from definition and measurement to the identification of demographic and psychological variables with which it correlates. The results reached by Romanian researchers illustrate that, overall, young Romanians consider themselves happy (Bernath Vincze, 2016). However, it was found that the levels of subjective happiness and satisfaction in life in adolescents in Romania were lower compared to European averages (Vincze et al., 2015). The authors state that these inequalities are mainly due to the lower socio-economic situation, the lack of adequate social cohesion, and the presence of risk factors in adolescents in Romania. Predictors of life satisfaction in young adults were the following: self-esteem, optimism, and illusory optimism about the future (Vincze and Szamoskozi, 2015).

Research studies point out that one of the most important factors that well-being is associated with is related to personality traits (Nishimura and Suzuki, 2016; Muntele Hendreş, 2021), in which personality traits being the most consistent predictors of SWB (Diener et al., 2003). Following the line of research on personality traits that may influence well-being, our study aims to highlight the role of openness and personal autonomy in the well-being of students in a pandemic.

#### Openness and Well-Being

Much discussed over the years, openness is the fifth personality factor in the Big Five model (along with extraversion, kindness, conscientiousness, emotional stability). Albu (2017) states that this personality trait has attracted the attention of many researchers, is known by various names, including *culture, intelligence, intellect, intellectance, fluid intelligence, care, attention, intellectual*, or *openness to experience*.

In the aforementioned study, the opening is approached in accordance with the beliefs of the author Albu (2017) and refers to the preference for diversity and non-conformism, to the existence of interests over intellectual and cultural aspects. Thus, the level of openness is measured by: the level of openness to learning, openness to the intellect, and openness in aesthetics.

Openness is relevant to students’ well-being during their university education, due to both: its relevance to the intellect (Bardi et al., 2009) and high academic performance (Vedel and Poropat, 2017; Gatzka, 2021). However, the results of field studies show data that do not indicate a direct association between openness and well-being. Some of the results of previous meta-analytical studies are pointed out by Bardi et al. (2009), the conclusion being that openness to experience is neither associated with satisfaction in life (e.g., DeNeve and Cooper, 1998; Heller et al., 2004; Steel et al., 2008), nor with reactions to stressful situations (e.g., Suls et al., 1998). Even if openness to experience was not inherently linked to well-being, this does not mean that the relationship between these variables could not be re-studied in specific contexts, where a high level of openness is required. For example, in situations where rapid change occurs, there are data that suggest that individuals with a high level of openness are more likely to accept the challenges posed by such situations (McCrae and Costa, 1985). This is due to the fact that people with high openness are characterized by a need for variety and may be motivated to actively seek out what is unfamiliar to them (McCrae, 1996). This less familiar context to students could be given by the very transition and adaptation to online education, in the situation given by the COVID-19 pandemic. In addition, field studies have shown a statistically significant correlation between openness to experience and extroversion with positive emotional mood (Shiota et al., 2007), a mood that can be understood as one of the components of SWB.

#### Personal Autonomy and Well-Being

Similar to other psychological constructs, personal autonomy has been defined in multiple ways (Albu, 2007, 2008) and differently approached. For example, the authors differentiate between autonomy as independence and autonomy as self-endorsed functioning (Chen et al., 2013). To conceptualize and measure the level of personal autonomy, we will follow the approach promoted in the Romanian literature, by Albu (2007, 2008) and Albu and Porumb (2009). Personal autonomy is a personality trait that consists of the ability to control one’s own life along with the feeling that there is a possibility to exercise this control (Albu, 2007; Albu and Porumb, 2009). In other words, autonomy refers to a person’s freedom to lead a life in accordance with his/her own desires and values (Sipos and Barratt, 2005). On the other hand, although in our study personal autonomy is considered a variable related to personality, in the theory of self-determination, Deci and Ryan (1985, 2000) define autonomy as a psychological need, along with the need for competence and affiliation. From this perspective, all individuals obtain well-being from the fulfillment of basic psychological needs (Deci and Ryan, 1985, 2000). Recent studies (Chirkov et al., 2003; Yu et al., 2018) capture the direct association between the need for autonomy and SWB in various settings and populations, including clinical samples, in family settings, in organizational work environments, or educational settings, regardless of the subjects’ gender. However, in our multidimensional study, personal autonomy is approached taking into account four dimensions: cognitive, behavioral, emotional, and value (according to Albu (2007), Albu and Porumb (2009)). Therefore, this is a variable related to personality; it is not confused with the fundamental need for autonomy, from the theory of self-determination (Albu, 2007; Albu and Porumb, 2009).

Field studies have concluded that autonomy is associated with life satisfaction and that it explains the substantial variation in SWB (Inglehart et al., 2008; Welzel and Inglehart, 2010). Furthermore, it is considered that autonomy, along with material concerns (income, financial satisfaction, and satisfaction to living standards), were important predictors of happiness and satisfaction in life (Ng, 2015).

In this study, we will consider the personal autonomy of students as a variable that is directly associated with their well-being but at the same time an independent variable (predictor), along with openness, in the regression analysis. The regression model in question will be able to capture the role of personal autonomy and openness to the students’ well-being in a pandemic. In addition, we want to underline the role of academic regulation mechanisms on well-being, aspects that we will refer to in the following.

### Well-Being and Academic Motivation

As already mentioned in the introductory part, our study is based on the theory of self-determination developed by Deci and Ryan (1985, 2000), mainly using motivations related ideas. We believe intrinsic motivation is extremely important for obtaining academic performance and well-being in the university, in the conditions given by this pandemic. Intrinsic motivation implies the highest level of behavioral self-regulation and personal autonomy. Intrinsically motivated people are characterized by curiosity, by the desire for cognitive emotional, and intellectual stimulation, getting involved in activities for sheer pleasure (Deci and Ryan, 1985, 2000). Studies conducted around the 1990s show that individuals who are intrinsically motivated tend to use strategies aimed at in-depth information processing; be creative; achieve better academic performance; report higher levels of well-being and self-esteem; be more confident in their own strengths and gain more satisfaction from the various activities they carry out throughout their lives (Ryan et al., 1997; Sheldon and Kasser, 1998). Recent studies also indicate that intrinsic motivation and amotivation are significant predictors of PWB among students who take online courses amid pandemics (Muzaffar and Yamin, 2021). But maintaining student motivation is already a challenge for teachers even in face-to-face environments and even more so, in distance learning (Goksel, 2021). On the other hand, some studies conclude that online learning, before the pandemic, had a positive effect on students’ motivation and academic performance (Harandi, 2015; Kumar and Bajpai, 2015). However, the research data of other studies show that the academic motivation of Pedagogy of Primary and Preschool Education students does not change, regardless of the teaching environment, i.e., online or face to face (Zwanch and Cribbs, 2021). The results of a study conducted on students in Romania and Lithuania show that students’ motivation for online learning in the pandemic is low to moderate, with intrinsic motivation being even lower than extrinsic motivation (Lamanauskas et al., 2021). The study conducted by Natalya and Halim (2021) is one that could better clarify the aspects related to the level of student motivation during the pandemic. The authors show that even if the academic motivation increased in the first days of the pandemic, 1 year after the implementation of online education, there was a significant decrease. However, researchers say that students can keep their intrinsic motivation high during COVID-19 isolation if they are interested in the subjects they are learning (Goksel, 2021) or if they have a high level of satisfaction with online courses (Algahtani et al., 2021). There are also some other research studies that have reached similar conclusions (see Popa and Bochiş, 2015), on groups of students in part-time education, emphasizing that an important role for student satisfaction with the hybrid training system is given by the quality of self-study materials and the quality of the relationship between peer students and teachers.

## The Current Research

In this hereby presented study, we aim to define a state of matter, of university education, in a pandemic, to underline the relationship between students’ well-being and internal psychological variables related to personality and mechanisms of regulation of academic motivation. Furthermore, we want to identify the answer to two questions. The first of these is: *How much of the variance in the level of self-perceived well-being of students in the pandemic can be explained by the level of openness and personal autonomy?* As mentioned before, openness and personal autonomy were conceptualized and measured as variables related to students’ personalities by Albu (2007, 2017) and Albu and Porumb (2009). The second question: *How much of the variance in the self-perceived well-being of students in the pandemic is explained by the mechanisms of regulating academic motivation, statistically controlling the impact of openness and personal autonomy?* The answer to the two questions should be given with the help of multiple regression analysis, trying to highlight the role of mechanisms to regulate academic motivation on the well-being of students in university, before the fourth wave of the pandemic. Although in previous research, personality traits have been kept under control to examine the correlation between the fulfillment of psychological needs and SWB (Sheldon and Niemiec, 2006; Nishimura and Suzuki, 2016), this study thoroughly uses existing information by underlining psychological variables that can influence students’ well-being of in a pandemic.

The main goal of this study: to point out the contribution of psychological factors, more precisely, the personality structure characterized by openness and personal autonomy, but also the mechanisms of regulation of academic motivation to explain the variance of results on the scale of self-perceived well-being of students in the university; thus, the following specific research hypotheses:

H_1_: *There is an association relationship between students’ self-perceived well-being in university and: their level of autonomy, their level of openness, and the mechanisms for regulating their academic motivation.*
H_2_: *Mechanisms for regulating academic motivation contribute significantly to explaining the variance of self-perceived well-being of university students, after eliminating the influence of personality-related variables: autonomy and openness.*

Please note that, due to the restrictions imposed by national and university policies, the students participating in the study had previously had three consecutive semesters only in online training (in synchronous and asynchronous activities) and when the research was conducted, their activity was still online.

## Materials and Methods

### Participants

The subject lot was of 150 students enrolled at the University of Oradea, Romania in undergraduate and master’s degree studies at the Faculty of Socio Humanistic Science, among which the majority were in undergraduate studies (72.66%) and Pedagogy of Primary and Preschool Education (62.01%). One of the peculiarities regarding the formation of student groups in the study programs of the Faculty of Socio Humanistic Science, University of Oradea, is the fact that most of the students are females. Additional data for the subject lot, according to the study program, year of study, background, gender, and age are shown in Table 1.

**TABLE 1:** The subject lot participating in the study (Descriptive statistics).

			*N*	%
**Faculty of Socio Humanistic Science**				
Pedagogy of Primary and Preschool Education	Undergraduate studies		93	62.01%
Special Psycho-pedagogy	Undergraduate studies		11	7.33%
Other study programs within the Faculty	Undergraduate studies		5	3.33%
Integrated Education in Pre and Primary Schools – MA	Master’s Degree		41	27.33%
			***N* = 150**	**100%**
Year of study	I		58	38.66%
	II		50	33.33%
	III		52	34.66%
			***N* = 150**	**100%**
Gender	Female		143	95.33%
	Male		7	4.66%
			***N* = 150**	**100%**
Social background	Urban		69	46%
	Rural		81	54%
			***N* = 150**	**100%**
Age	Minimum	Maximum	Mean	Standard deviation
	18.00	52.00	26.75	9.722

### Instruments

The research tools used had been previously validated on the Romanian student population which are the following: *Personal autonomy questionnaire*, *Openness questionnaire*, *Questionnaire for measuring the types of regulation of academic motivation*, and *Inventory of students’ self-perceived well-being*.

***Personal autonomy questionnaire*** was designed under the coordination of Monica Albu (Albu, 2007; Albu and Porumb, 2009), having 36 items. It consists of four scales, one for each dimension of personal autonomy: cognitive autonomy (nine items; e.g., *I like to make decisions on my own, without depending on others*), behavioral autonomy (11 items; e.g., *I do as I think it is better, without taking into account the opinion of others*), emotional autonomy (eight items; e.g., *I express my feelings without hesitation, even if others do not agree with them*), and value autonomy (8 items; e.g., *I stick to my principles in any situation*). For each item, the subject had to decide the degree to which the statement was true for him/her and choose one of the options, “very little,” “little,” “not too much, not too little,” “much,” and “very much.” For the whole questionnaire, the coefficients of internal consistency presented by the author of the test in the validation study had values higher than 0.75 (being between 0.752 and 0.846). Also, in terms of validity, at all questionnaire scales were obtained high saturations in the extracted factor (linear correlations between scales and factor are significant at the threshold *p* < 0.001). In the present study, the alpha Cronbach coefficient for the whole scale was high, of 0.910, and for the subscales, it was between 0.803 and 0.943.

***Openness questionnaire*** was developed, validated, and calibrated on the Romanian population by Albu (2007). It consists of 18 items that evaluate three dimensions of Openness: openness to learning (seven items; e.g., *I am interested in constantly improving my professional skills.*), openness in aesthetics (five items; e.g., *I believe there should be more buildings dedicated to culture and art.*) and the ideatic opening (six items; e.g., *I like scientific challenges.*). For each item, the subject had to decide the degree to which the statement was true for him/her and choose one of the options, “very little,” “little,” “not too much, not too little,” “much” and “very much.” According to the author, in the validation study, there was a good internal consistency for all scales, the coefficient α having values between 0.763 and 0.860 and a good validity of the instrument (Albu, 2007). In our study, the coefficient α for the whole scale was high, of 0.918, and on subscales, it was between 0.897 and 0.921.

***Questionnaire for measuring the types of regulation academic motivation*** was used in studies on the Romanian population by Robu and Tufeanu (2020). The tool is an adapted version of the *Questionnaire of the types of regulation of school activity* designed and validated by Drugaş (2009), by making use of the theory of self-determination. In our study, it contains 21 items, grouped in four subscales: intrinsic adjustment of motivation (seven items), adjustment of identification motivation (four items), external adjustment of motivation (seven items), and amotivation (three items). The subjects had to choose an answer on a 5-level Likert scale, ranging from 1 – total disagreement to 5 – total agreement. Here are some examples of items related to each subscale. Intrinsic motivation: *I enjoy doing the activities required by the study program I am enrolled in*; *Graduating the program of study and the faculty is important to me*. Identification motivation: *I feel that the study program I am enrolled in is helping me to develop as a person*, or *I feel that my academic study program is developing skills that I will use later in life*. Intrinsic motivation: *I rarely think about the grades I will get* (reverse scoring); *It is important for me that others appreciate what I do in college*; and, the examples of items for amotivation: Apart from the fact that I have something to fill my time with, I *see no reason to continue this study program*; *I don’t think the study program I am enrolled in is worth graduating*.

For the sample group of students who participated in the study conducted by Robu and Tufeanu (2020), the values of internal consistency, *a* Cronbach, were satisfactory, with values between 0.67 and 0.74. In this hereby presented research, a satisfactory value was obtained on the internal consistency of the items at the global score, α Cronbach = 0.724, and values between 0.71 and 0.75 per subscale.

***Inventory of students’ self-perceived well-being*** was designed based on the tool designed by Adams et al. (1997) to identify the well-being felt at the individual level in several areas of life. The inventory includes 36 statements to which the subject responds on a 6-level Likert scale (from “strong agreement” to “strong disagreement”), indicating the level of agreement with each statement. For our study, the variant adapted and validated on the Romanian population was used by Roşeanu and Răşcanu (2008). According to the authors, the subscales of the research tool are positive attitude toward the self, environment, and life (14 items), negative attitude toward the self, environment, and life (13 items); self-perceived physical health (seven items). The items were revised or rephrased to identify the well-being of university students during the pandemic. Thus, the first two subscales were renamed as positive/negative attitudes toward self, others (peers and teachers), and student life. Examples of items for the subscale of positive attitude toward self, others, and student life are the following: *In general, I felt confident in my ability to learn in college*; *My friends and other peers relied on my help*; *The intellectual challenges I faced in college were vital to my overall well-being*. Examples of items for the subscale Negative attitude toward self, others and student life: *There were times when I felt inferior to most of my peers*; *I didn’t see many perspectives in my student life for the future*; *My life as a student often seemed void of positive mental stimulation*. Examples of items for the Physical Health subscale are the following: *My body seems to cope with physical illness very well*; *Compared to other fellow-colleagues, my physical health has been excellent*. The internal consistency of the α Cronbach items has values between 0.75 and 0.84 on the subscales (Roşeanu and Răşcanu, 2008). In our study, α Cronbach coefficients had values between 0.77 and 0.85.

### Procedures

The students’ subject lot entered the research based on consent, informed, and anonymous. The administration of the research toolkit took place in the second half of October when, after 2 weeks after returning to the mixed training system (online courses and on-site seminars and labs), it was decided to switch to an exclusively online system for 3 weeks, due to the increase in the incidence of the number of active cases of coronavirus infection (COVID-19), in Romania and Bihor county. The questionnaires were administered online, the average fill-in time was of 30 min.

### Data Analyses

The research data were analyzed with the SPSS version 20. After removing the incomplete information from the database, the research hypotheses were tested. The correlation between well-being and personality traits (openness and personal autonomy), on one hand, and between student well-being and academic regulation mechanisms, on the other hand, was achieved with the help of the Pearson test as data distribution had been symmetrical. In order to explore the explanatory value of the mechanisms for regulating motivation (independent variable) for students’ well-being (dependent variable), the hierarchical regression analysis was performed. Before entering the research data into the regression analysis, it was checked whether the database contained influential cases (using the “Cook’s Distance” method), whether there were multi-collinearity issues, and whether the assumption of independent variables was verified. We can state that the database does not contain influential cases, wherein for none of the independent variables included in the model, there were no multi-collinearity problems, the values of *Tolerance* coefficient being higher than 0.50. Furthermore, for variance inflation factor (VIF), the values did not exceed 1.78. The assumption of independent errors was fulfilled with the value Durbin-Watson = 1,829. Finally, examination of the normal P-P graphic of the residual standardized regression of positive attitude toward self, others, and student life indicates a normal distribution.

## Results

### Correlation Between Students’ Self-Perceived Well-Being and Personal Autonomy, Openness, and Mechanisms for Regulating Motivation

To test the correlation between the dimensions of students’ self-perceived well-being and personality-related variables, on the one hand, and the mechanisms of motivation adjustment, on the other hand, Pearson correlation was used.

Research data presented in Table 2 indicate the correlation between the research variables and the effect sizes ranging from low to high. There were high and average effect size correlations between self-perceived well-being expressed through a positive attitude toward self, others, and student life, and some of the subscales of the research tools. Thus, according to study data presented in Table 2, positive attitude toward self, others, and student life correlates directly with: behavioral autonomy (*r* = 0.428, *p* < 0.001, moderate effect size), cognitive autonomy (*r* = 0.546, *p* < 0.001, strong effect size); openness to learning (*r* = 0.541, *p* < 0.001, strong effect size), aesthetic openness (*r* = 0.488, *p* < 0.001 moderate to high effect size); intrinsic motivation (*r* = 0.541, *p* < 0.001, strong effect size), identification motivation (*r* = 0.411, *p* < 0.001, moderate effect size).

**TABLE 2:** Pearson correlation between students’ self-perceived well-being and personal autonomy, openness, and mechanisms for regulating motivation.

Variables		Students’ self-perceived well-being		
		
		Positive attitudine	Negative attitudine	Physical health
Autonomy of values	Pearson correlation	0.326**	–0.180*	0.162*
	Sig. (two-tailed)	0.000	0.027	0.048
	*N*	148	150	149
Emotional autonomy	Pearson correlation	0.219**	–0.457**	0.103
	Sig. (two-tailed)	0.008	0.000	0.210
	*N*	148	150	149
Behavioral autonomy	Pearson correlation	0.428**	–0.161*	0.282**
	Sig. (two-tailed)	0.000	0.049	0.000
	*N*	148	150	149
Cognitive autonomy	Pearson correlation	0.546**	–0.229**	0.311**
	Sig. (two-tailed)	0.000	0.005	0.000
	*N*	148	150	149
Openness to learning	Pearson correlation	0.541**	–0.039	0.331**
	Sig. (two-tailed)	0.000	0.640	0.000
	*N*	148	150	149
Ideatic openness	Pearson correlation	0.360**	–0.196*	0.197*
	Sig. (two-tailed)	0.000	0.016	0.016
	*N*	148	150	149
Aesthetic openness	Pearson correlation	0.488**	–0.032	0.182*
	Sig. (two-tailed)	0.000	0.697	0.026
	*N*	148	150	149
Intrinsic motivation	Pearson correlation	0.541**	–0.108	0.363**
	Sig. (two-tailed)	0.000	0.190	0.000
	*N*	148	150	149
Identification motivation	Pearson correlation	0.514**	–0.245**	0.283**
	Sig. (two-tailed)	0.000	0.003	0.000
	*N*	148	150	149
External motivation	Pearson correlation	0.031	0.279**	–0.072
	Sig. (two-tailed)	0.705	0.001	0.386
	*N*	148	150	149
Amotivation	Pearson correlation	–0.109	0.267**	–0.037
	Sig. (two-tailed)	0.188	0.001	0.656
	*N*	148	150	149

**p < 0.01, *p < 0.05.

At the same time, we conclude that self-perceived well-being in terms of negative attitude toward self, others, and student life, on one hand, and health, on the other hand, is associated with the other variables having a low effect size, with a few exceptions. These exceptions are for the correlation between the negative attitude toward self, others and student life, and emotional autonomy (reverse correlation, *r* = –0.457, *p* < 0.001 moderate to high effect size); the association between physical health and cognitive autonomy (*r* = 0.311, *p* < 0.001, moderate effect size), openness to learning (*r* = 0.331, *p* < 0.001, moderate effect size); intrinsic motivation (*r* = 0.363, *p* < 0.001, moderate effect size).

### Importance of Variables Related to the Mechanisms for Regulating Motivation, Personal Autonomy, and Openness in Explaining the Variance of Students’ Well-Being in a Pandemic

We used hierarchical regression analysis to underline the contribution of mechanisms of academic motivation regulation on the PWB of students in university, in terms of statistical control over variables related to personal autonomy and openness. However, as pointed out in the previous study, in testing specific hypothesis 1, there were significant correlations only between certain research variables, so the second hypothesis of the study was revised and rephrased. In regression models, we would introduce only the variables that in the previous study correlated with a moderate or high effect size. Thus, the positive attitude toward self, others, and student life was included as a dependent variable, and the dimensions it correlated with became the independent variables of the study. So, the factors introduced in model 1 are part of the category of personality traits, such as cognitive and behavioral autonomy (the mean of the two subscales was calculated) and the openness to learning and aesthetics (the mean of the two subscales was calculated). Further on, in Model 2, we kept under control the factors introduced in Model 1, in order to isolate the influence of mechanisms of academic motivation regulation, i.e., intrinsic motivation and identification (mean of the two subscales), on the dependent variable.

The research data for the two models tested in hierarchical regression analysis showed statistically significant results (*F*_change_ = 43,615, *p* < 0.01, for the first model, and *F*_change_ = 14,411, *p* < 0.01, for the second model). As shown in Table 3, positive attitude toward self, others, and student life has a significantly high correlation (*p* < 0.001) with all three independent variables.

**TABLE 3:** Descriptive statistics and correlation matrix for research model variables.

Variables	Mean	Standard deviation	*N*	1	2	3
Positive attitude toward self, others, and student life	4.846	0.885	148	0.510**	0.568**	0.558**
(1) Autonomy (behavioral and cognitive)	3.713	0.560	148	–	0.280**	0.439**
(2) Openness (to learning and aesthetics)	4.245	0.626	148		–	0.485**
(3) Motivation (intrinsic and identification)	4.121	0.657	148			–

**p < 0.001.

Next, the testing of each variable within the two models was performed using the Student’s *t*-test, the results being statistically significant for each independent variable included in the two steps of the hierarchical multiple regression (Table 4).

**TABLE 4:** Hierarchical multiple regression analysis of explanatory factors of well-being (positive attitude toward self, others, and student life).

*Variables*	*R* ^2^	*R^2^adjust.*	*Beta*	*B*	*SE b*
**Step 1**	0.456**	0.449**			
Behavioral and cognitive autonomy			0.381**	0.602	0.457
Openness to learning and aesthetics			0.461**	0.652	0.101
**Step 2**	0.488**	0.472**			
Behavioral and cognitive autonomy			0.286**	0.452	0.113
Openness to learning and aesthetics			0.350**	0.495	0.106
Academic motivation			0.231**	0.365	0.135

**p < 0.01, *p < 0.05.

#### First Step

The explanatory model contained the independent variables: personal and cognitive autonomy, along with openness to learning and aesthetics, which together explain 45.6% of the variance of positive attitude toward self, others, and student life (*R*^2^ = 0.456). After eliminating the variance due to the other variable included in the model, a direct correlation was obtained between the positive attitude toward self, others, and student life and cognitive and behavioral autonomy (β = 0.381, *p* < 0.001). Similar results were obtained for the correlation between the positive attitude toward self, others, and student life and the openness to learning and aesthetics (β = 0.461, *p* < 0.001).

Eliminating the simultaneous influence of independent variables on the dependent variable, the value of semi-partial correlation coefficients showed, for the opening variable, a specific coefficient of determination of 26% (*r*_part_ = 0.515) and for the autonomy variable, a specific coefficient of determination of 19.3% (*r*_part_ = 0.444) on the dependent variable.

Thus, the openness to learning and aesthetics, and cognitive and behavioral autonomy were variables that explained the positive attitude of students toward self, others, and student life in the pandemic.

#### Second Step

The independent variables from step 1 are controlled, and in the second, intrinsic and identification motivation was introduced. In this case, the model was statistically significant, and together with the three independent variables could explain 48.8% of the variance of positive attitude toward self, others, and student life (*R*^2^ = 0.488, *p* < 0.01). Compared to Model 1, the increased value was not particularly important (*R*^2^ = 0.042, *p* < 0.01), but being statistically significant, we could consider that the positive attitude of students toward self, others, and student life could be explained by the mechanisms of academic motivation regulation. In addition, at the analytical level, at the final estimation model, the values of the coefficient β were statistically significant for all three variables included in the model: openness, autonomy, and academic motivation, as shown by the data in Table 3.

Eliminating the simultaneous influence of independent variables on the dependent variable, the value of the semi-partial correlation coefficients on the dependent variable showed a specific coefficient of determination of 16.5% (*r*_part_ = 0.407) for openness, a specific coefficient of determination of 12% (*r*_part_ = 0.353) for autonomy, of 7% (*r*_part_ = 0.278) for intrinsic and identification motivation.

Research data underline the idea that beyond certain personality traits (openness, autonomy), the mechanisms of self-regulation of intrinsic and identification motivation have a significant influence on self-perceived well-being. These data emphasize once again the need to stimulate and increase students’ interest, intrinsic and identification motivation for university training for future professions.

## Discussion

Unlike previous studies, this current research is based on the theory of self-determination to analyze the role of regulation mechanisms of students’ academic motivation on their well-being in the pandemic, when personality variables (openness and personal autonomy) are controlled. Research results validate the results of previous studies, i.e., students’ well-being is associated with their personality (Inglehart et al., 2008; Welzel and Inglehart, 2010; Nishimura and Suzuki, 2016; Muntele Hendreş, 2021) and with their academic motivation (Deci and Ryan, 1985, Ryan and Deci, 2000; Ryan et al., 1997), emphasizing that the association between these variables is present even in conditions of unexpected and problematic change in education, such as the pandemic context.

Among the three analyzed dimensions of students’ self-perceived well-being in university (positive and negative attitudes toward self, others and student life, physical health), only the positive attitude toward self, others, and student life is associated with moderate or high effect sizes, with most of the subscales of the research tools (Table 2). Thus, specific Hypothesis 1 is partially validated.

The first of the psychological variables expressed in Table 2 as being associated with a positive attitude toward self, others, and student life and correlated to the positive well-being toward self, others and student life was personal autonomy, as a personality trait; as concluded by other researchers (e.g., Chirkov et al., 2003; Inglehart et al., 2008; Welzel and Inglehart, 2010; Yu et al., 2018), the correlation was statistically significant. Furthermore, our study shows that students’ positive attitude is directly associated (with a moderate to a high effect size) with cognitive and behavioral autonomy (Table 2). In addition, a negative attitude toward self, others, and student life has a reverse association with the level of emotional autonomy. A possible explanation: personal autonomy is a trait that is grouped around other social dispositions such as introversion, locus of internal control, intrinsic motivation, self-confidence/arrogance, non-conformism, desire for solitude, and asocial or antisocial learning (Feist, 1999). Consequently, the fact that the very cognitive and behavioral autonomy leads to a positive attitude of students in pandemics, as autonomy could intensify individual study in the asynchronous environment, favors the fulfillment of work tasks and the achievement of personal goals independently, without requiring continuous support from outside.

The second of the psychological variables, openness, as a personality trait, is directly related to a positive attitude toward self, others, and student life. The statistically significant results of the correlation between positive attitude toward self, others and student life, and openness on two of its dimensions: to learning and aesthetics, were also the most surprising for us because, in similar studies, different results had been presented (see for example DeNeve and Cooper, 1998; Heller et al., 2004; Steel et al., 2008; Bardi et al., 2009). However, the premise from which we have drawn our first specific hypothesis stated that the level of openness can be associated with well-being under certain specific conditions such as those that require a high level of openness to adapt to rapid changes (McCrae and Costa, 1985). A further explanation: openness to experience is associated with positive emotional moods (Shiota et al., 2006), and consequently, a more positive attitude toward life. Therefore, even if the period of isolation in the pandemic was most intensely felt by extroverts (Ivantchev and Stoyanova, 2021), the results of our study show that students with high levels of openness and personal autonomy have a positive attitude toward self, others, and student life.

The third psychological variable, one being associated with a positive attitude toward self, others and student life is academic motivation, or more exactly, the mechanisms for regulating academic motivation. Continuing the line of research based on the theory of self-determination of Deci and Ryan (1985), Ryan and Deci (2000), the present study underlined the association between positive attitude toward self, others, and student life with two of the regulating mechanisms of academic motivation, i.e., intrinsic motivation and identification motivation (Table 2). Similar results have been obtained in other studies conducted before the pandemic. For example, Sheldon et al. (2004; cited in Secu, 2021) and Rudy et al. (2007) considered that, alongside intrinsic motivation and identification motivation, it is associated with a higher state of well-being. We believe, that even after three consecutive semesters online, students who managed to maintain their intrinsic and identification motivation, also showed a more positive attitude toward self, others, and student life.

Although there were interesting results in testing hypothesis 1, the focus of our research fell on the analysis of personality variables, so as to show the contribution of regulation mechanisms of motivation on students’ well-being in university, in the pandemic (specific Hypothesis 2). The method used in data processing was hierarchical multiple regression, conducted for explanatory purposes to analyze the factors with a significant influence on the positive attitude of students toward self, others, and student life. Research results in the multiple regression analysis point out that all the variables included in the regression model explain the dispersion of the results of students’ well-being, with an effect size around moderate values. There were slightly higher effect sizes for independent or predictor variables related to personality (openness and autonomy) and slightly lower for academic motivation (intrinsic and identification). However, we consider that research data presented in Table 4 show that the level of intrinsic regulation of motivation and identification significantly contributes to explaining the level of variance of students’ well-being in the pandemic, going beyond their personality. For explanatory purposes, we shall give an example. If students have high scores on the predictor variables (openness, autonomy), we can say that in the conditions under which students simultaneously have the following (Albu, 2007; Albu and Porumb, 2009): (1) high level of cognitive autonomy – i.e., a high capacity to make decisions on one’s own, to critically analyze the received information, to have opinions without being influenced by others, to self-evaluate; (2) high level of behavioral autonomy – i.e., it acts according to his/her own beliefs, without taking into account the opinions of others, does not abandon a task when difficulties are encountered, strives to manage on his/her own; (3) high level of openness to learning – i.e., a high interest in personal development and lifelong learning; (4) high level of aesthetic openness – i.e., interest in art and beauty; and all these levels are controlled, the variations in intrinsic and identification motivation will influence students’ well-being in the pandemic. This has multiple implications, especially in school and professional counseling, educational psychology, and even positive psychology. Although it has been found that in the pandemic, the interest in learning has increased only in extroverted students (see Smith et al., 2021), our findings show that not only personality but also regulating mechanisms of academic motivation will influence students’ well-being. That is why it is extremely important that career decisions of young people should be in accordance with their intrinsic interests and mechanisms, and identification with specific academic activities of university education. These variables will significantly contribute to maintaining a positive attitude toward self, others, and life in the face of rapid changes that may occur, in critical situations or in crises in education, or even in their future professions.

## Conclusion

The following lines will discuss the implications of our findings, strengths and limitations, and directions for future research.

The research contributes to the consolidation of a long series of research in positive, personality, and educational psychology, highlighting the role of variables related to personality and academic motivation in providing students’ well-being in the pandemic. The study of psychological variables involved in maintaining people’s well-being has been studied before, but without continuity in the research direction undertaken by us. For example, in studies that used advanced data processing statistical methods, the role of personality variables as mediators in the correlation between well-being and level of satisfaction of basic psychological needs was highlighted (Schüler et al., 2016). However, in order to complete such results, personality variables and academic regulation mechanisms were taken into account in the present study, allowing for a deeper understanding of the way students kept their well-being toward self, peers, and teachers and to student life, in the pandemic.

This is interdisciplinary research. It uses the theory of self-determination and other empirical data and results of field research that investigate well-being and psychological variables associated with it, but in a more particular context, i.e., in the pandemic and of students. As shown by the results of the studies mentioned in the introductory part, in the pandemic, students encountered several difficulties in the transition and adaptation to online education (see for example, Yusuf and Jihan, 2020; Aftab et al., 2021; Huang and Zhang, 2021). We consider that some of these obstacles could be overcome with the help of some internal psychological variables, related to personality and regulation mechanisms of student motivation. For example, previous studies have found that in the pandemic, extrovert students, compared to introvert ones, showed a greater interest in learning (Smith et al., 2021).

Our research data suggest that students may keep a positive attitude toward themselves, others, and student life during the pandemic, if they show openness and personal autonomy, and/or if they have intrinsic and identification motivation in academic learning activities. Perhaps, the most important conclusion of this study is that intrinsic and identification motivation will contribute to the positive attitude toward self, others, and student life in the pandemic, even when personality variables are controlled. The followers of Deci and Ryan (1985), Ryan and Deci (2000) emphasized the role of intrinsic motivation for a successful activity. However, the findings of previous studies are contradictory in terms of students’ motivation levels before and during the pandemic period. For example, some studies conclude that students’ academic motivation remains at the same level, regardless of the environment in which the courses are conducted, online or on-site (Zwanch and Cribbs, 2021); in others, it is said that academic motivation is lower in online (Lamanauskas et al., 2021), or that, on the contrary, students’ motivation is higher in online (Malinauskas and Požėrienė, 2020), but also that it fluctuates in online during the pandemic period (Goksel, 2021). Furthermore, the literature and theory of self-determination, the basis of our study (Deci and Ryan, 1985, Ryan and Deci, 2000), analyzed the role of intrinsic motivation in obtaining well-being, but there is the necessity of further research to identify new regulation mechanisms of students’ academic motivation in the pandemic, coping with the transition and adaptation to a less familiar learning environment, i.e., synchronous and asynchronous. Our findings show that both motivations, intrinsic and identification, have an important role in maintaining a positive attitude toward self, others, and student life in the pandemic. By explaining the variance of students’ well-being in the pandemic depending on their level of academic motivation, the general implications of the research are significant. We believe that while pandemic isolation has had a negative impact on the social relationships with other colleagues and teachers, students’ openness, personal autonomy, and academic motivation can determine a positive attitude toward self, others, and student life, maybe even more in isolation than on ordinary basis.

### Practical Implications

The basic idea of this research will be useful for teachers, psychologists, and school counselors and why not, even for students, so they could outline a school career in accordance with the activities they value, but also to use their personal resources in the purpose of overcoming difficulties or obstacles that arise in learning. Its findings primarily provide the necessary premises for the development of educational practices that support pupils and students in overcoming obstacles in learning activities. We believe the new model of education initiated in the pandemic, although pursuing the same purposes and contents as in the traditional system, must pay more attention to the values and attitudes promoted in the academic courses, targeting those that contribute to maintaining the well-being of university students. As Laurian-Fitzgerald and Fitzgerald (2021) point out, it is time for teachers and students to take control of their own learning, as education needs to be transformed and aligned with the realities of the 21st century through active and continuous student involvement. To this, it is added the fact that education must be carried out in a more pleasant environment for the current generation of students, based on modernism and actuality (Muntean, 2021).

Overall, we believe there is a need to stimulate students, in frontal activities, openness to experience and learning or curiosity for knowledge, by valuing their interests in activities based on differentiated teaching. Then, in independent activities, teachers can encourage the cognitive and behavioral autonomy of students, taking also into account other of their personality traits, which are associated with academic performance, such as conscientiousness (see Bochiş and Florescu, 2018). Students will develop cognitive and behavioral autonomy when they feel encouraged to explore, take initiative, and implement solutions to their problems (Niemiec and Ryan, 2009). Another important aspect for teachers and specialists in the Sciences of Education is to be aware that if they build a culture of classroom autonomy, students develop their intrinsic motivation (Niemiec and Ryan, 2009). Lack of autonomy, on a motivation level, too, together with the persistence of extrinsic motivation in students (e.g., They learn for grades, praise, or approval from others) does not contribute to maintaining well-being, in situations when the support of others (teachers, colleagues, or parents) can no longer be given the same measure. Certainly, increasing students’ level of openness, autonomy, and intrinsic and identification motivation are the key issues that need to be considered by teachers and school counselors, not only in the university but especially throughout the entire school life of a student. Our research findings could be of interest to both employers and future graduates, especially for those who, after completing their studies, choose to work in areas where there are rapid situation changes, when they will have to continuously improve themselves in order to overcome the difficulties at work, with the purpose of keeping their SWB. Therefore, the efforts of teachers in educational institutions and school counselors should be directed toward addressing the methods that increase the level of intrinsic and identification motivation, thus helping to maintain a positive attitude toward self, others, and student life, even in difficult situations. At the same time, effective collaboration between various specialists in the sciences of education, psychology, youth mental health, and government could help minimize and mitigate the negative impact of the COVID-19 pandemic on the SWB of pupils and students. Such a goal was pursued at a university in the UK for psychology students and the program led to gratifying results as students were introduced to key concepts related to well-being and resources to help increase it (Morgan and Simmons, 2021).

### Research Limitations and Future Research Paths

Our study has several limitations that need to be analyzed, and a first thing can be connected to the selection of study participants, mostly females. However, the limit is justified by the fact that the participants were selected from specializations followed mainly by female graduates [see for example the data presented by Popa et al. (2015), related to the particularities and structure of groups of students in the Pedagogy of Primary and Preschool Education, Faculty of Socio Humanistic Sciences, University of Oradea]. Another limitation is that the research could have had a mixed, explanatory, and predictive purpose, but the small number of subjects allowed only an explanatory research design. Consequently, the research data serve only to explain the factors that influence the positive attitude toward self, others, and student life of the subject lot. Therefore, in future research, we should consider including a larger sample, a balanced distribution of respondents according to gender. However, we believe that important results have been achieved in this area. Diener and Lucas (2000 cited in Muntele Hendreş, 2021) believe that in order to develop positive psychology in a country, it is necessary to research and know the specifics of SWB in that culture so that subsequent interventions can be made taking into account the results of local research. In addition, as we pointed out in the results and discussions section, this study emphasizes that SWB is associated with personality traits and intrinsic motivation, such results being found in similar research, from which we designed the basis of our research.

Another limitation is that the socio-demographic variables of the subject lot were not included in the study, although it is known that well-being is the expression of the balance between individual characteristics (e.g., personality predispositions or acquired skills throughout life) and environmental factors (e.g., socioeconomic status, family, social relations, and professional career). The results of pandemic studies have indicated that age, sex, marital status, socio-economic status, and chronic health conditions are predictors of emotional well-being during the COVID-19 pandemic (Al Mutair et al., 2021). However, future studies can successfully complete the results of our study, which is intended to be a favorable starting point for researchers who want to explore only the psychological variables associated with well-being during the periods of adaptation of students to new learning environments, along with the rapid changes taking place in the knowledge society.

## Data Availability Statement

The raw data supporting the conclusions of this article will be made available by the authors, without undue reservation.

## Ethics Statement

The studies involving human participants were reviewed and approved by the Ethics Committee for Research, Faculty of Socio-Humanistic Sciences, University of Oradea. The participants provided their informed consent to participate in this study.

## Author Contributions

LB: conceptualization, methodology, and writing – original draft preparation. LB, KB, and MF: software, formal analysis, investigation, and writing – review and editing. LB and MF: visualization. All authors contributed equally to the article and approved the submitted version.

## Conflict of Interest

The authors declare that the research was conducted in the absence of any commercial or financial relationships that could be construed as a potential conflict of interest.

## Publisher’s Note

All claims expressed in this article are solely those of the authors and do not necessarily represent those of their affiliated organizations, or those of the publisher, the editors and the reviewers. Any product that may be evaluated in this article, or claim that may be made by its manufacturer, is not guaranteed or endorsed by the publisher.
